# Network analytics and machine learning for predicting length of stay in elderly patients with chronic diseases at point of admission

**DOI:** 10.1186/s12911-022-01802-z

**Published:** 2022-03-10

**Authors:** Zhixu Hu, Hang Qiu, Liya Wang, Minghui Shen

**Affiliations:** 1 School of Computer Science and Engineering, University of Electronic Science and Technology of China, No. 2006, Xiyuan Ave, West Hi-Tech Zone, 611731 Chengdu, Sichuan People’s Republic of China; 2 Big Data Research Center, University of Electronic Science and Technology of China, Chengdu, People’s Republic of China; 3 Health Information Center of Sichuan Province, Chengdu, People’s Republic of China

**Keywords:** Length of stay, Machine learning, Multimorbidity network, Network analysis, Patient similarity network, Point of admission

## Abstract

**Background:**

An aging population with a burden of chronic diseases puts increasing pressure on health care systems. Early prediction of the hospital length of stay (LOS) can be useful in optimizing the allocation of medical resources, and improving healthcare quality. However, the data available at the point of admission (PoA) are limited, making it difficult to forecast the LOS accurately.

**Methods:**

In this study, we proposed a novel approach combining network analytics and machine learning to predict the LOS in elderly patients with chronic diseases at the PoA. Two networks, including multimorbidity network (MN) and patient similarity network (PSN), were constructed and novel network features were created. Five machine learning models (eXtreme Gradient Boosting, Gradient Boosting Decision Tree, Random Forest, Linear Support Vector Machine, and Deep Neural Network) with different input feature sets were developed to compare their performance.

**Results:**

The experimental results indicated that the network features can bring significant improvements to the performances of the prediction models, suggesting that the MN and PSN are useful for LOS predictions.

**Conclusion:**

Our predictive framework which integrates network science with data mining can forecast the LOS effectively at the PoA and provide decision support for hospital managers, which highlights the potential value of network-based machine learning in healthcare field.

## Background

With a rapidly aging population, the incidence of chronic diseases has increased dramatically, which imposes serious social and economic burdens on countries around the world [1, 2]. It has been estimated that over 75% of the elderly have more than one chronic condition [3]. Multimorbidity in old age (i.e., the co-existence of two or more chronic diseases in one individual) has become a prominent problem worldwide, resulting in greater medical demands, greater healthcare utilization, and higher cost [4, 5]. Early prediction of length of stay (LOS) for patients with chronic diseases, especially the elderly with multimorbidity, can help hospital managers to allocate limited resources, control patient costs effectively, and improve the quality of medical services [6, 7]. Early prediction has therefore received increasing attention from researcher.

Machine learning has been widely applied to forecast the LOS due to its outstanding nonlinear fitting ability and superior predictive ability. Xie et al. [8] developed a bagged regression trees model to predict the LOS using insurance claim data and found that the medical data (e.g., diagnosis codes) contributed more to the LOS predictions than demographic data. Daghistani et al. [9] adopted Random Forest (RF), Artificial Neural Networks (ANNs), Support Vector Machine (SVM), and Bayesian Network (BN) to forecast the LOS for cardiac patients. Their results indicated that the RF has the best performance and good interpretability. To date, despite the use of a growing number of machine learning models to forecast LOS, most studies have focused on patients with specific diseases, such as heart failure [10], cardiovascular disease [11], and strokes [12], which limit their practicability and scope of application.

Moreover, relatively few studies have predicted LOS at the point of admission (PoA), which is more meaningful than later clinical stages, because it can provide an essential component for service and resource planning in patient and family counseling [13]. The prediction of LOS at the PoA is a challenging task due to the limited data available at such an early stage of treatment. Typically, at the PoA, the inpatient ward has limited data such as the primary patient information, hospital characteristics, and diagnostic data (i.e., principal diagnosis and comorbid conditions). Due to the value of diagnostic data for forecasting LOS at the PoA, prior studies have attempted to use features extracted from the Charlson Comorbidity Index (CCI) and Elixhauser Comorbidity Index (ECI) to predict LOS [14, 15]. However, CCI and ECI only cover a limited number of diseases, which did not take full advantage of all the diagnostic information available, resulting in limited powers of LOS prediction. Furthermore, some studies have tended to ignore the historical hospitalization data on patients, which is a significant factor in predictive models [6, 16, 17]. Extracting features from patients’ historical records may improve the performance of LOS prediction models [18, 19].

So far, growing evidence shows that comorbid conditions have a significant impact on the LOS [10, 20, 21]. However, how to reasonably transform comorbid conditions into features is still a challenge for researchers. Simply encoding comorbid conditions as features using one-hot encoding would generate thousands of features and result in the curse of dimensionality and expensive training time. Recent research into network medicine development provides a new approach to understanding the complex interrelations between diseases. In a Phenotypic Disease Network, also known as a Disease Co-occurrence Network (DCN) [22], links between diseases are based on their significant co-occurrence. Such networks provide an overview of the co-occurrence of multiple conditions in a network structure and have been used to study the multimorbidity patterns underlying depression [23], heart failure [24], and chronic obstructive pulmonary disease (COPD) [25]. Meanwhile, some researchers have tried to use the results of DCN analysis for predictive purposes. Srinivasan et al*.* [26] extracted two network features from DCN (high-cost propensity and community membership scores) and applied tree-based models to predict high-cost patients. They found that the network features could significantly improve the model’s performance. Xu et al*.* [27] developed a Diagnoses to Vector model (Dx2Vec) based on DCN to predict individual self-harm behavior. Their results showed that the DCN could excavate multimorbidity patterns and further enhance the model’s accuracy. Sideris et al*.* [28] clustered the DCN constructed from Electric Health Record (EHR) data to reduce the data dimensionality and applied the cluster information as features to predict diabetes readmission prediction. The results indicated that, compared with CCI and ECI features, the predictive accuracy was improved by 4.65–5.75% using network features. However, few studies have explored the predictive ability of disease network features in LOS prediction, and the potential has yet to be excavated.

Moreover, the Patient Similarity Network (PSN), where nodes represent patients and edges represent the similarities between pairwise patients, has also received extensive attention in recent years. Valuable features are extracted through the PSN using network analysis for various health prediction tasks. Lu et al*.* [29] constructed a PSN using disease co-occurrence and extracted node centrality to predict the risk of type 2 diabetes mellitus (T2DM). Guo et al*.* [30] created a generic framework called Patient similarity based on Domain Fusion (PsDF), which performs patient similarity assessments on each available domain data separately, and then integrates the affinity information over various domains into a comprehensive similarity metric. Their experimental results showed that the PsDF facilitated prediction of outcome of incident cases of end stage kidney disease (ESKD) and severe aortic stenosis (AS). Therefore, we propose to extract valuable features from the PSN to predict the LOS.

In this paper, we contribute to the existing body of knowledge by developing a novel approach to forecasting the LOS for elderly patients with chronic diseases at the PoA using network analytics and machine learning models. The main contributions are summarized as follows:

1. A predictive framework combining Multimorbidity Network (MN), PSN, and machine learning was proposed to predict hospital LOS at the PoA.
2. A space-friendly and high-efficiency development algorithm was presented for constructing MN on large datasets.
3. A PSN was constructed that utilizes the label information from a patient’s neighbors to enrich the feature representation of patients.

To the best of our knowledge, this is the first study that integrates MN and PSN with data mining models to effectively predict LOS in elderly patients with chronic diseases at the PoA. Our proposed approach is not only suitable for predicting LOS in elderly patients with a single disease, but also for patients with multimorbidity, which expands the scope and practical ability of the model. We believe that our predictive framework has a universal scope and can be used in other health prediction areas.

## Methods

### Overview of the research framework

**Fig. 1:**
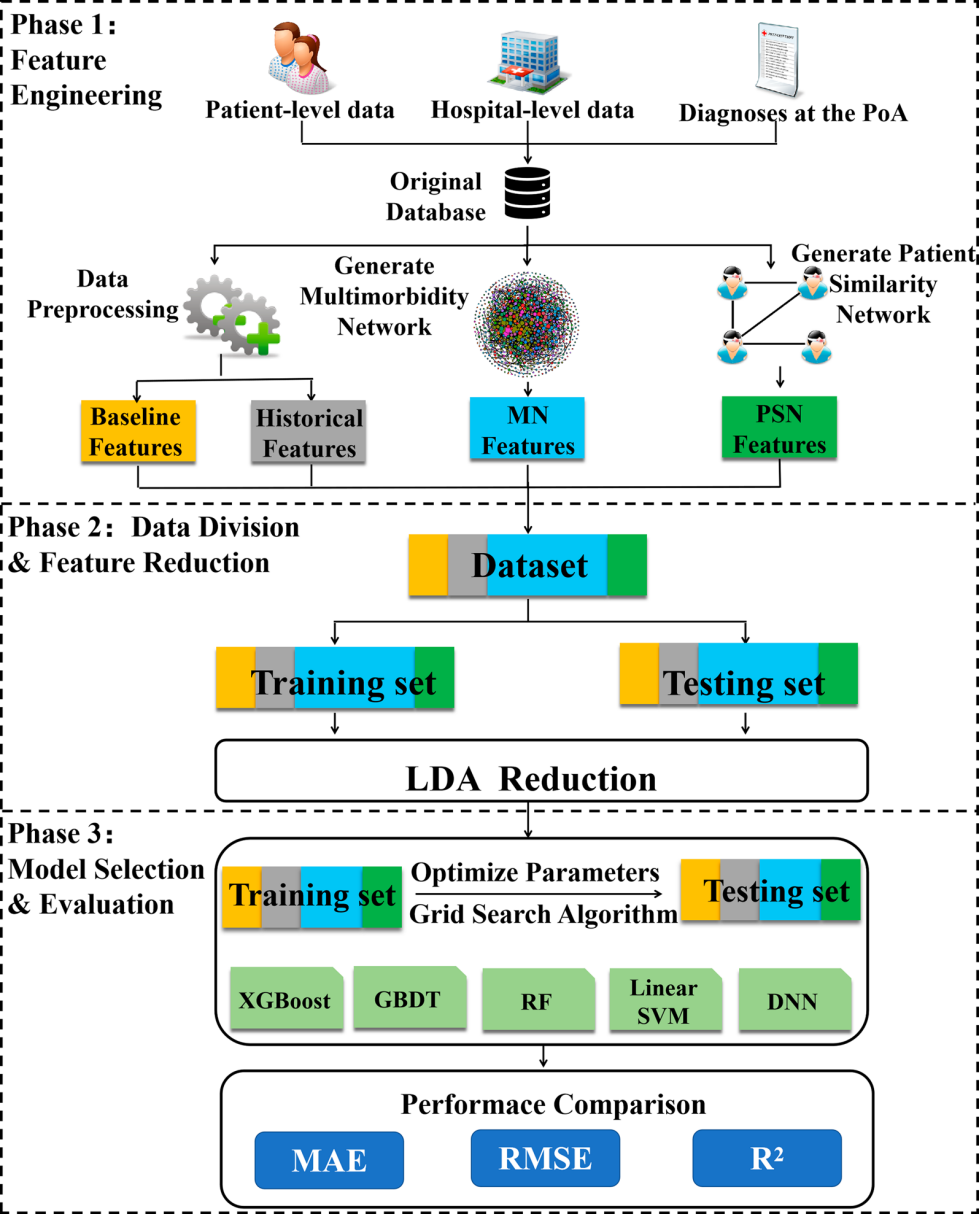
Flowchart of proposed predictive model

An overview of the current study is shown in Fig. 1. Our methods can be summarized as follows. The first phase involved obtaining the original dataset, including patient-level data, hospital-level data, and the diagnoses at the PoA. An MN and a PSN were then developed for feature engineering. Four feature sets, including the baseline, historical, MN and PSN features, were extracted to form the modeling dataset. In the second phase, the dataset was randomly split into a training set (80%) and testing set (20%). Reduction via Linear Discriminant Analysis (LDA) was performed to compress MN features as much as possible while retaining the most representative information. In the third phase, we applied five machine learning models to predict LOS. These were: eXtreme Gradient Boosting (XGBoost), Gradient Boosting Decision Tree (GBDT), RF, LinearSVM, and Deep Neural Network (DNN). A grid search was utilized to seek optimal model parameters and select the best models on the training set. Subsequently, we applied mean absolute error (MAE), root mean square error (RMSE), and coefficient of determination (R^2^) to evaluate the models’ performances on the testing set.

### Data

In the present study, we used the Hospital Discharge Records (HDR) for the urban areas of Chengdu, China, which contains 10.7 million records from 678 hospitals and covers the period from January 1, 2015 to December 31, 2019. Each record consists of patient-level data (e.g., anonymized identity, age, sex, the date of admission, and the date of discharge), hospital-level data (e.g., hospital level, hospital address, and hospital affiliation), discharge diagnoses (including a principal diagnosis and up to 15 secondary diagnoses), and the corresponding flag variable which represents whether the disease is diagnosed at the PoA. All diagnoses are specified by the ICD-10 codes (International Classification of Diseases, 10th Revision) at the three-digit level. The present study was approved by the Ethics Committee of the Health Information Center of Sichuan Province. The requirement of obtaining informed consent was waived because of the secondary nature of the de-identified data in the retrospective study design.

In order to meet the requirements of the present study, several inclusion criteria were applied as follows. (1) The LOS was not null, and the date of admission was between 2015 and 2019. (2) The patient was alive during the 2015 to 2019 period. (3) The age of the patient was 65 years or older. (4) To eliminate the outliers of the LOS, we regarded those LOS which were greater than a 99% quantile (58 days) as the outliers. Hence, the LOS needed to be less than or equal to 58 days. (5) Since the MN only included chronic diseases, each record must have at least one chronic disease at the PoA. The criteria for judging the chronic diseases came from a previous study [31]. For the patients aged 65 years or older in our dataset, there were 685 chronic diseases in total. The proportion of chronic diseases and the average number of chronic diseases were 95.56% and 6.5 respectively, indicating that the patients suffered from a heavy burden of chronic diseases. Finally, 2.5 million hospitalization records and about 1.1 million individuals were retained. Descriptive statistics on the main variables are shown in Table 1. The data from 2015 to 2017 were used to construct the MN and the data from 2018 to 2019 were applied to develop the PSN and our predictive models.

**Table 1:** Descriptive statistics of main variables in our dataset

Category		Counts (proportion)	Mean (std) of the LOS
Total	–	2,543,758 (100.00%)	12.3 (7.5)
Gender	Male	1,237,624 (48.65%)	12.4 (7.6)
	Female	1,306,134 (51.35%)	12.2 (7.4)
Years	2015	260,745 (10.25%)	13.2 (8.0)
	2016	423,586 (16.65%)	12.6 (7.6)
	2017	550,686 (21.65%)	12.4 (7.5)
	2018	610,795 (24.01%)	12.2 (7.5)
	2019	697,946 (27.44%)	11.9 (7.4)
Age group	65–69	594,045 (23.35%)	11.8 (7.3)
	70–74	645,257 (25.37%)	12.1 (7.3)
	75–79	575,939 (22.64%)	12.3 (7.3)
	80–84	416,941 (16.39%)	12.6 (7.5)
	85–89	228,725 (8.99%)	13.4 (8.4)
	90 +	82,851 (3.26%)	13.9 (9.1)
Ethnic group	Han	2,533,984 (99.62%)	12.3 (7.5)
	Minority	9,774 (0.38%)	12.6 (7.8)

**Fig. 2:**
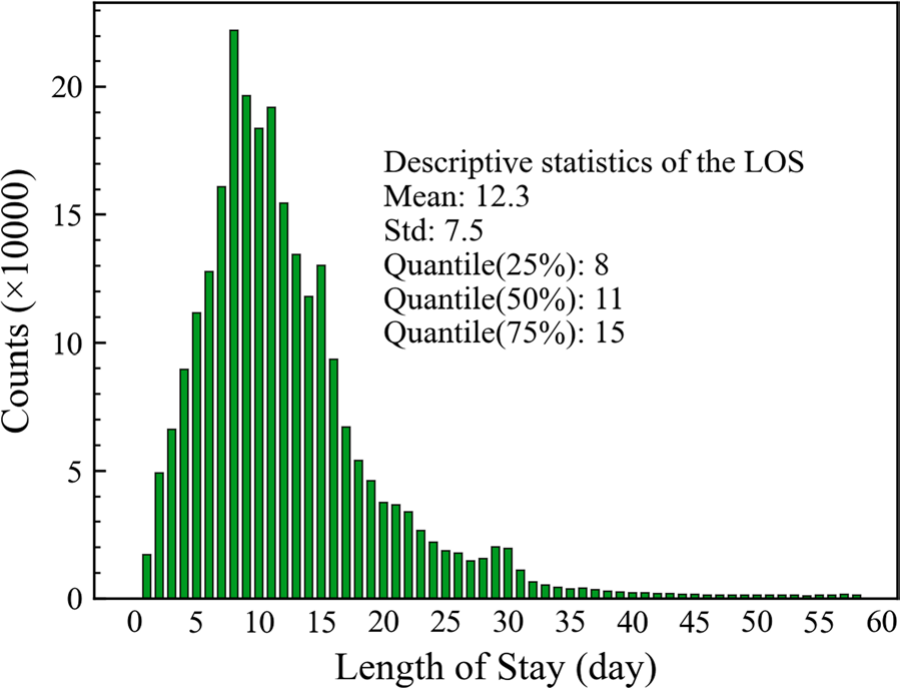
Distribution and descriptive statistics of the LOS

Due to the time of admission or discharge is only exact to the day in our dataset, we defined the LOS as the number of days between the admission date and discharge date. The distribution of the LOS is shown in Fig. 2. Almost 50% of the LOS are between 8 and 15 days, and the mean of the LOS is about 12.3 days, which is much higher than previous studies due to the elderly having a more extended LOS.

### Multimorbidity network construction

The MN was developed based on 1,235,017 hospitalization records and 661,324 individuals from 2015 to 2017, so the MN is a kind of DCN and only contains chronic diseases. The Relative Risk (RR) was adopted to measure the distance among comorbidities. The RR of observing a pair of diseases *i* and *j* affecting the same patient is given by Eq. (1).

1 $$RR_{ij} = \frac{{C_{ij} *N}}{{C_{i} *C_{j} }}$$

In Eq. (1), the *C*_*ij*_ is the number of patients with both diseases, *N* is the total number of patients in the population, and *C*_*i*_ and *C*_*j*_ are the numbers of patients with disease *i* and disease *j* [22]. The 99% confidence intervals of RR were estimated using the Katz et al. method [32], as shown in Eqs. (2) and (3).

2 $$\left[ {RR_{ij} \times \exp \left( { - 2.58\sigma_{ij} } \right),RR_{ij} \times \exp \left( {2.58\sigma_{ij} } \right)} \right]$$

3 $$\sigma_{ij} = \frac{1}{{C_{ij} }} + \frac{1}{{C_{i} C_{j} }} - \frac{1}{N} - \frac{1}{{N^{2} }}$$

**Fig.3:**
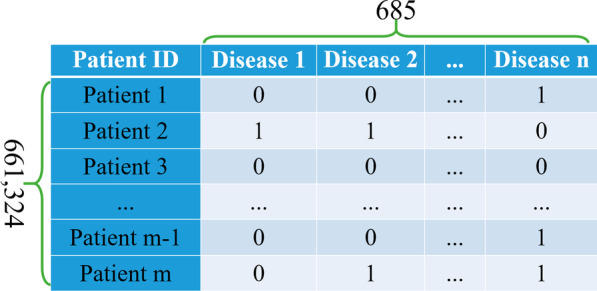
Patient-disease matrix (entries indicate whether a patient has a disease)

**Fig. 4:**
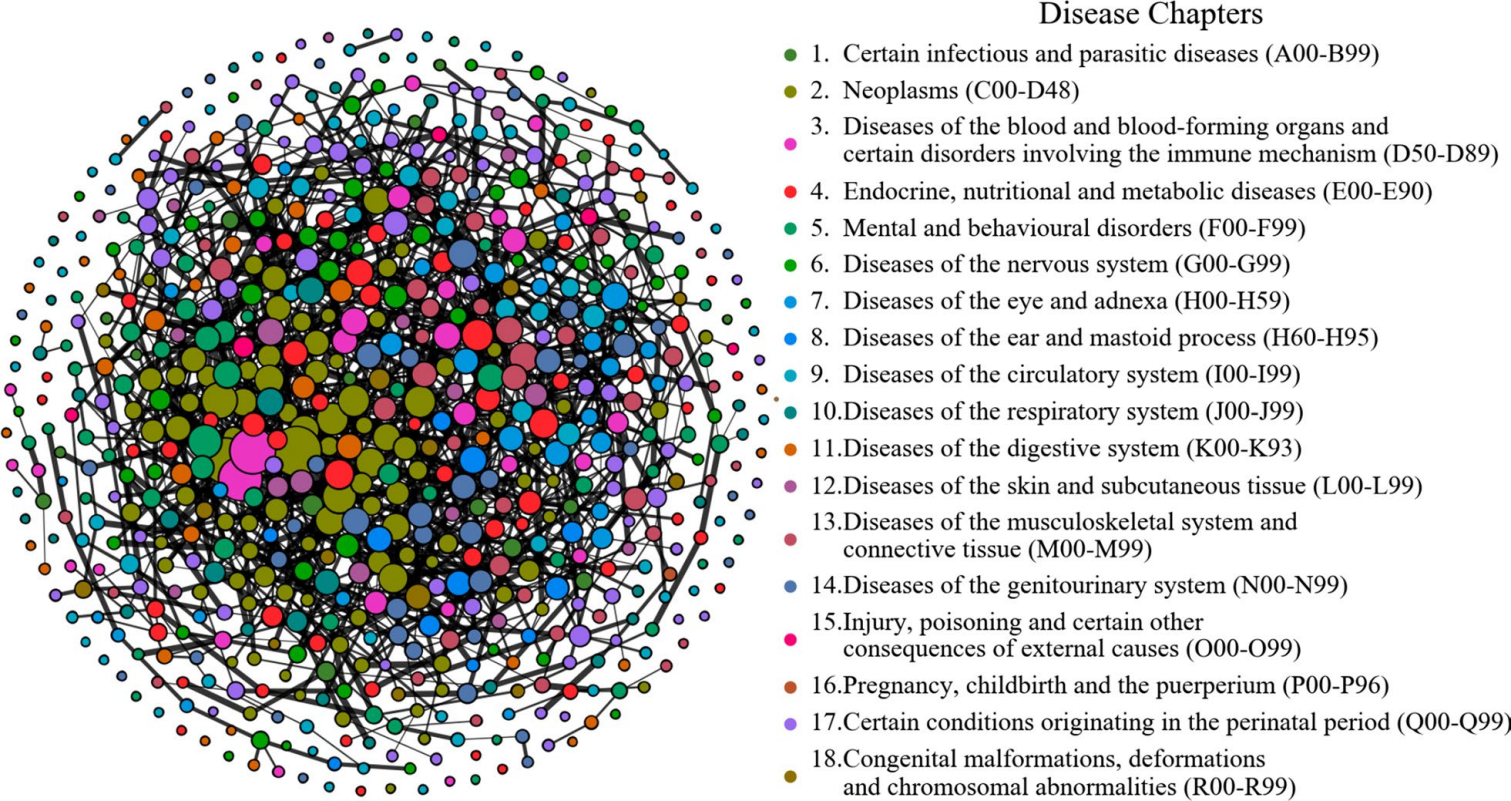
Visualization of the MN (only the edges with RR greater than 30 are reserved for visualization purpose). The nodes represent chronic disease; The colors of nodes represent 18 disease chapters according to the ICD-10, and the size of nodes is positively correlated with the degree of nodes; The edges represent the co-occurrence relationship between pair-wise diseases


For convenience, a two-dimensional patient-disease matrix was constructed to calculate the RR, as shown in Fig. 3. It is almost impossible to load such a massive matrix with 661,324 rows and 685 columns into computer memory, which makes it harder to construct an MN on large datasets. Also, the density of the matrix is only 0.86%, which results in much spatial redundancy. To address this problem, we performed some space optimization and efficiency optimization to make it easier to build the MN on large datasets, as summarized in Algorithm 1. Sparse matrix technology was applied as the compressed sparse column matrix (*csc_matrix)* is efficient for column slicing and matrix multiplication. There are some other efficiency optimization details worth noting, such as using matrix multiplication in line 12 rather than counting directly the co-occurrences of pairwise diseases, which can save time when the dimensions of the matrix are very high. After applying Algorithm 1, an undirected weighted MN with 683 nodes and 35,860 edges was obtained. A network visualization is shown in Fig. 4. The MN can represent the complex and implicit inner relationships between diseases that may or may not appear at the PoA. For example, a patient was diagnosed with essential hypertension (I10) at the PoA. The diagnoses during the hospitalization were chronic kidney disease (N18) and type 1 diabetes mellitus (E10). Such co-occurrence relationships can be easily found and quantified in the MN, and can be extracted as features to help predict downstream tasks.

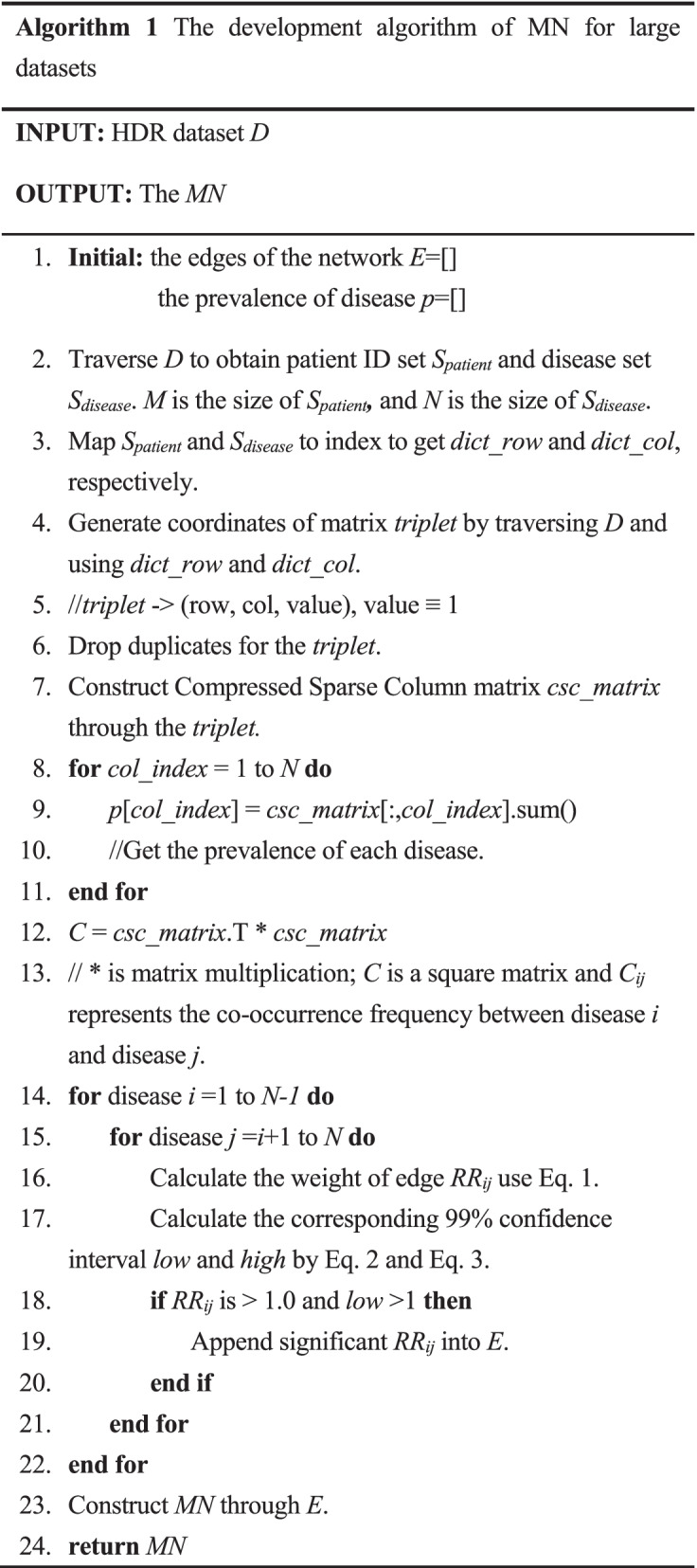


### Patient similarity network construction

Experimental results of the Unified Message Passaging model (UniMP) [33] have shown that considering the label information on neighbors can bring significant improvements to prediction tasks. Therefore, inspired by the label propagation method of UniMP, we constructed a PSN to excavate extra information for the LOS prediction, with the assumption that similar patients would have similar LOS in the network.

In the PSN, nodes represent patients (training samples) and edges represent the similarities between patients. We used Jaccard index to measure the similarity, as defined in Eq. (4).

4 $$similarity\left( {i,j} \right) = \frac{{\left| {d\left( i \right) \cap d\left( j \right)} \right|}}{{\left| {d\left( i \right) \cup d\left( j \right)} \right|}}\;\;where\;g\left( i \right) = g\left( j \right) \& \& a\left( i \right) = a\left( j \right)$$

**Fig. 5:**
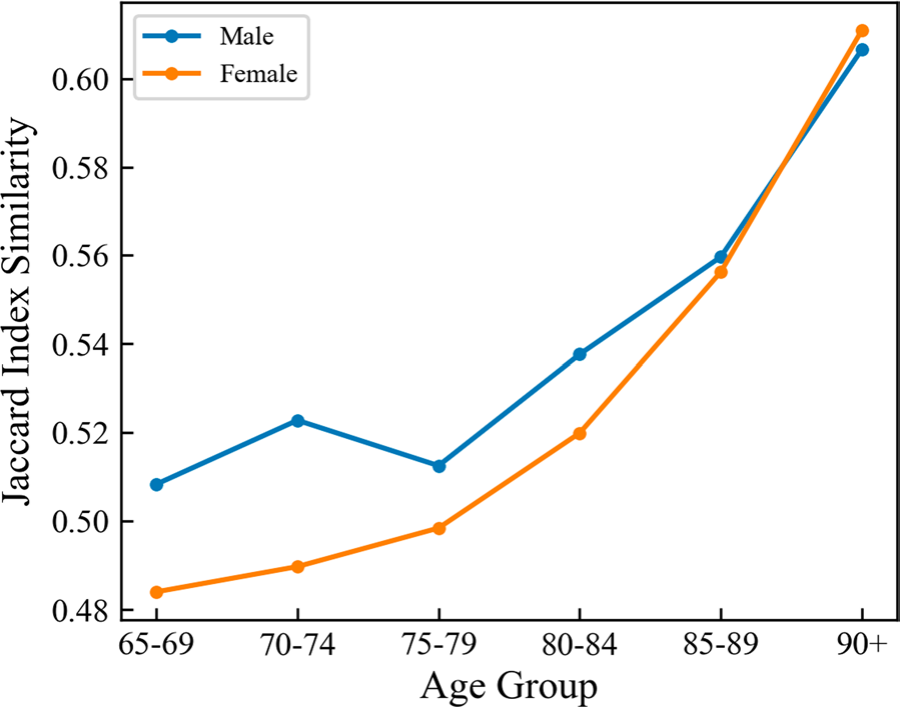
The average Jaccard index similarity in each group. The similarity of the female is generally less similar than that of the male. The similarity increased with age, because the older the patient, the more diseases the patient has

In Eq. (4), patient $i$ and patient $j$ must be of the same gender $g\left( \cdot \right)$ and the same age group $a\left( \cdot \right)$ (as shown in Table 1), while $d\left( i \right)$ represents the diagnosed diseases of patient $i$ at the PoA. The similarity network considers gender, age and disease information to evaluate patient similarity comprehensively. For each patient, however, we only consider the most similar 100 neighbors in the training set because of the impossibility of calculating all potential similarities among millions of patients. We therefore applied an approximate nearest neighbor algorithm called NMSLIB [34] to conduct heavy kNN computations, which can use an approximation algorithm to find k nearest neighbors with superior recall and queries per second accelerate [35]. The method took only 15 min to find the 100 most similar neighbors for each patient in the entire dataset. The average similarity in each group is shown in Fig. 5.

### Feature engineering

In order to make full use of the limited data available, four feature groups were extracted from the existing data. These were the baseline features, historical features, MN features, and PSN features. Detailed information on feature extraction and feature grouping are shown in Table 2. The baseline features make full use of the patient, hospital, and date information. The diagnostic information at the PoA was extracted as the ECI related features. This is a method generally applied in the literature to quantify diagnostic information. The historical features were extracted to indicate the histories of the patients’ physical health. Two kinds of features, eigenvector centrality features and disease risk features, were extracted from the MN. The PSN features are derived from the PSN.

**Table 2:** Feature descriptions

Feature name	Descriptions	Types^a^	Number
Baseline features			69
Date features	The year, month, and day of the week of admission	N	3
Gender	Male or Female	D	2
Age	Age of the patient	N	1
Hospital affiliation	The affiliation of the hospital	N	1
Admission status	1. Danger 2. Urgent 3. General	N	1
Patient's and Hospital's address code	The smaller the value, the closer to the city center	N	2
Address flag	Whether the patient's address code is equal to the hospital's address code	N	1
Hospital levels	Measuring hospital quality	N	2
Number of diseases	Number of diseases at the PoA	N	1
Hospital admission source	1. Emergency treatment 2. Outpatient service 3. Transferred from Other medical institutions 4. Others	D	4
Ethnic group	Han or minority	D	2
Job	The occupation of the patient	D	13
Marital status	1. Spinsterhood 2. married 3. Divorce 4. Missing	D	4
Elixhauser comorbidity index [36]	Including AIDS HIV, alcohol abuse, blood loss anemia, and so on	D	31
Elixhauser comorbidity score [37]	A mapping score to represent one's health condition	N	1
Historical features			8
Descriptive statistics of historical LOS	Extract the counts, mean, standard deviation, median, min, and a max of these LOS	N	6
Last discharge interval	The days between the last discharge date and the date of current admission	N	1
Last LOS	The LOS of the last hospital admission	N	1
MN features			657
Eigenvector centrality features	For each chronic disease in the MN, extracting its eigenvector centrality value as features	N	653
Disease risk features	Extract the counts, maximum, mean, and sum of disease risk scores	N	4
PSN features			5
Descriptive statistics of neighbor's LOS	Extract the mean, standard deviation, median, min, and a max of these LOS	N	5

^a^The N and D represent the numerical feature and discrete feature, respectively. One-hot encoding will be used for the discrete features

#### Eigenvector centrality (EVC) features

The EVC score is a measure attribute of diseases (nodes) in the MN, representing the importance of the disease and influenced by neighbor diseases. For a given graph $G:\left( {V,E} \right)$ with $\left| V \right|$ vertices, let $A = \left( {a_{v,t} } \right)$ be the adjacency matrix. The relative centrality $x$ as the score of vertex $v$ can be defined as Eq. (5).

5 $$x_{v} = \frac{1}{\lambda }\mathop \sum \limits_{{t \in {\mathcal{N}}\left( v \right)}} x_{t} = \frac{1}{\lambda }\mathop \sum \limits_{t \in G} a_{v,t} x_{t}$$

In Eq. (5), ${\mathcal{N}}\left( v \right)$ is a set of the neighbors of $v$ and $\lambda$ is a constant. With a small rearrangement this can be rewritten in vector notation as the eigenvector equation Eq. (6).

6 $$Ax = {\uplambda }x$$

The eigenvector $x_{max}$ corresponding to the largest eigenvalue $\lambda_{max}$ is the EVC. The $v{\text{th}}$ component of the $x_{max}$ then gives the relative centrality score of the vertex $v$ in the network. Hence, we can obtain a EVC score for each disease in the MN. Finally, the EVC scores were transformed as EVC features. For example, considering that a patient may have several chronic diseases at the PoA, multi-hot encoding was used to map these diseases to a vector (which only contains one and zero, and the one represents the corresponding observed disease). Then, the one was replaced with the EVC score that uniquely corresponds to the disease. Overall, 653 EVC features were obtained. It is worth noting that the number of EVC features is less than the number of network nodes since some diseases do not appear in 2018 and 2019.

#### Disease risk features

In the MN, different diseases have different effects on LOS. We therefore use disease risk features to quantify the differences between diseases. The mean of the LOS of the disease can be calculated by Eq. (7).

7 $$los\_mean\left( v \right) = \frac{1}{{\left| {\{ p_{j} |d\left( v \right) \in D\left( {p_{j} } \right)\} } \right|}}*\mathop \sum \limits_{{p_{i} \in \{ p_{j} |d\left( v \right) \in D\left( {p_{j} } \right)\} }} LOS\left( {p_{i} } \right)$$

In Eq. (7), $v$ is a node in the MN; $d\left( v \right)$ is the disease corresponding to node $v$; $p_{i}$ and $p_{j}$ represent different patients; $D\left( {p_{j} } \right)$ is the disease set of the patient $p_{j}$ at the PoA, and the $LOS\left( {p_{i} } \right)$ is the LOS of the patient $p_{i}$. Disease risk score and features can then be obtained by Eqs. (8) and (9).

8 $$disease\_risk\_score\left( v \right) = EVC\left( v \right)*los\_mean\left( v \right)$$

9 $$disease\_risk\_feature\left( {p_{i} } \right) = agg\left\{ {disease\_risk\_score\left( v \right){|}d\left( v \right) \in D\left( {p_{i} } \right)} \right\}$$

In Eq. (8), $EVC\left( v \right)$ is the EVC of node $v$. The disease risk score takes into account both the EVC of the disease and the influence of the disease on LOS. In Eq. (9), a patient may have several chronic diseases as the PoA, and several aggregation functions $agg\left( {*} \right)$ are used to quantify the distribution of the patient's disease risk scores, which include counts, maximum, mean, and summation functions.

#### PSN features

Based on the assumption that similar patients would have similar LOS, we can also extract statistics of the neighbors’ LOS as features from the PSN, as shown in Eq. (10).

10 $$PSN\_feature\left( {p_{i} } \right) = agg\left\{ {LOS\left( {p_{j} } \right){|}p_{j} \in {\mathcal{N}}\left( {p_{i} } \right)} \right\}$$

In Eq. (10), given a patient, 100 neighbors can be found from the PSN, and their LOS can be also obtained. Several $agg\left( * \right)$ functions, including the mean, standard deviation, median, min, and max functions, were used to extract statistics of the neighbors’ LOS.

There were a few missing values among some features. Therefore, we used zero for the missing value. For these category features, we regarded them as numerical features if there were ordinal relations between them, which could significantly reduce the features’ dimensions. Otherwise, one-hot encoding was used to encode the category features. To ensure that the time window for feature extraction was consistent with the historical features, we only looked back on the past three years of historical records. For instance, the date of admission of one patient was June 1, 2018. Those records whose dates of admission were between June 1, 2015 and May 31, 2018 were considered to represent the patient’s historical records. Some patients (nearly 35%) may not have any admission records during the past three years; zero was then adopted as the missing value. The EVC features were calculated from the MN, and LDA reduction was applied to decrease the feature dimensions. In addition, five features of the neighbor’s LOS were obtained from the PSN to provide additional information to aid the LOS forecast.

### Linear discriminant analysis

As the numbers of EVC features were up to 653, feature reduction was performed to save computational time, reduce information noise, and retain representative information as much as possible. LDA, as proposed by Fisher [38], is a supervised method that computes the linear discriminant by maximizing the distance between classes and minimizing the distance within classes [39]. If the dataset has *K* classes, the LDA can reduce dimensions up to *K* − 1. In this paper, the LOS is discrete and LDA can be applied to reduce dimensionality. We used the validation set’s performance to select the best number of dimension reductions; the best number has the lowest evaluation error in the validation set. In a preliminary experiment, we reduced the dimensions of EVC features from 653 to 32 by using LDA. We used the reduced EVC features to complete the following experiments.

### Model

To evaluate the proposed framework, we compared the performances of five machine learning models which used five different feature subsets. The five combinations of input features were as follows: *Baseline*, *Baseline* + *History*, *Baseline* + *MN*, *Baseline* + *PSN*, and *Baseline* + *History* + *MN* + *PSN*. The five machine learning models were XGBoost [40], GBDT [41], RF [42], LinearSVM [43], and DNN. To evaluate the framework’s accuracy and efficiency on our large-scale dataset, the five machine learning models were applied to predict the LOS.

The XGBoost, GBDT, and RF models are tree-based ensemble models, which have superior nonlinear fitting ability, robustness, and interpretability. As for SVM, we did not use a radial basis function kernel since it is inefficient and unsuitable to apply to millions of datasets. We therefore chose a linear kernel as the kernel function for the SVM. Standard normalization was adopted for the datasets before training the LinearSVM model.

DNN is also a common model in the LOS area [9]. The network architecture can highly affect the generalization ability of the model. In the current research, we tried several architectures and finally chose an architecture of six layers with “118-400-200-100-50-1”. The activation function was ReLU, and mean square error was adopted as the loss function. We used an Adam optimizer with lr = 0.0005 to train our model, with the weight decay set to 0.00001. Batch normalization and dropout were used to avoid model overfitting. The batch size was 4096 and epochs were 200, the numbers being determined by the grid search strategy.

To evaluate the generalization abilities of the five models, the dataset was randomly split into a training set (80%) and a testing set (20%). Since there were more than a million samples in the training set, we randomly divided 20% of them to form a validation set, which was applied to evaluate model performance in the parameter tuning process using the grid search strategy. All experiments used Python 3.7.3 on a Linux server with 48 Intel Xeon E5-2678 processors. A Pandas 0.24.2 toolbox and an sklearn 0.23.0 toolbox were used for data preprocessing and model training, respectively. Torch 0.3.0 was applied to train the DNN model.

### Evaluation

To evaluate the performance and generalization abilities of the different models, MAE, RMSE, and R^2^ were used, as shown in Eqs. (11), (12), and (13), where $y$ and $\hat{y}$ are the observed and the predicted LOS, respectively; $y_{mean}$ is the mean of $y$; and $n$ is the size of the dataset. The MAE and R^2^ are standard metrics that have been widely used in the LOS prediction task. The MAE show the average deviation between the observed and the predicted values. The R^2^ indicate whether the model is better than the mean forecast model; the more excellent the value of R^2^, the better the performance of the model. Multiple criteria can help to create a comprehensive evaluation of a model’s generalization performance.

11 $$MAE\left( {y,\hat{y}} \right) = \frac{{\mathop \sum \nolimits_{i = 1}^{n} \left| {y_{i} - \hat{y}_{i} } \right|}}{n}$$

12 $$RMSE\left( {y,\hat{y}} \right) = \sqrt {\frac{{\mathop \sum \nolimits_{i = 1}^{n} \left( {y_{i} - \hat{y}_{i} } \right)^{2} }}{n}}$$

13 $$R^{2} \left( {y,\hat{y}} \right) = 1 - \frac{{\mathop \sum \nolimits_{i = 1}^{n} (y_{i} - \hat{y}_{i} )^{2} }}{{\mathop \sum \nolimits_{i = 1}^{n} (y_{i} - y_{mean} )^{2} }}$$

## Results

### Comparison of models on different feature subsets

The reduced EVC features were obtained after the LDA reduction. Parameter tuning using grid search was then applied to each model to select optimal hyper-parameters. The comparison of the predictive performances of XGBoost, GBDT, RF, LinearSVM, and DNN on different feature subsets are listed in Table 3. The R^2^ of our proposed approach (*Baseline* + *History* + *MN* + *PSN*) was 0.375 for XGBoost, 0.374 for GBDT, 0.347 for RF, 0.285 for LinearSVM, and 0.330 for DNN, suggesting that the XGBoost outperforms the other models. For the other feature subsets, the R^2^ of XGBoost was 0.250 for *Baseline*, 0.316 for *Baseline* + *History*, 0.304 for *Baseline* + *MN*, and 0.316 for *Baseline* + *PSN*, which means using historical features or MN features or PSN features alone could significantly enhance model performance compared with the *Baseline*. Furthermore, the *Baseline* + *History* + *MN* + *PSN* experimental results show the best performance for all the models under all the evaluation criteria compared with the performance of other feature subsets. Notably, the R^2^ of XGBoost on *Baseline* + *History* + *MN* + *PSN* was improved by 18.7% compared with the R^2^ of XGBoost on *Baseline* + *History*, indicating that adding network features bring significant improvements to the model’s performance.

**Table 3:** The comparison of predictive performance of XGBoost, GBDT, RF, LinearSVM, and DNN on different feature subsets

Models	Metrics	Baseline	Baseline + History	Baseline + MN	Baseline + PSN	Baseline + History + MN + PSN
XGBoost	MAE	4.528 ± 0.006	4.276 ± 0.007	4.300 ± 0.007	4.241 ± 0.007	4.024 ± 0.006
	RMSE	6.419 ± 0.013	6.130 ± 0.015	6.182 ± 0.013	6.128 ± 0.013	5.859 ± 0.013
	R^*2*^	0.250 ± 0.002	0.316 ± 0.002	0.304 ± 0.001	0.316 ± 0.001	0.375 ± 0.002
GBDT	MAE	4.531 ± 0.007	4.280 ± 0.006	4.306 ± 0.006	4.251 ± 0.009	4.026 ± 0.006
	RMSE	6.422 ± 0.014	6.136 ± 0.013	6.189 ± 0.012	6.139 ± 0.013	5.861 ± 0.011
	R^2^	0.249 ± 0.002	0.314 ± 0.002	0.302 ± 0.001	0.314 ± 0.001	0.374 ± 0.001
RF	MAE	4.553 ± 0.008	4.343 ± 0.007	4.343 ± 0.006	4.297 ± 0.008	4.106 ± 0.007
	RMSE	6.468 ± 0.014	6.229 ± 0.015	6.256 ± 0.013	6.226 ± 0.014	5.987 ± 0.015
	R^2^	0.238 ± 0.002	0.293 ± 0.002	0.287 ± 0.002	0.294 ± 0.001	0.347 ± 0.002
Linear SVM	MAE	4.982 ± 0.007	4.697 ± 0.006	4.714 ± 0.006	4.571 ± 0.007	4.366 ± 0.006
	RMSE	7.004 ± 0.011	6.622 ± 0.013	6.710 ± 0.011	6.549 ± 0.012	6.265 ± 0.013
	R^2^	0.107 ± 0.001	0.201 ± 0.002	0.180 ± 0.001	0.219 ± 0.001	0.285 ± 0.001
DNN	MAE	4.595 ± 0.053	4.371 ± 0.043	4.390 ± 0.036	4.302 ± 0.043	4.152 ± 0.046
	RMSE	6.518 ± 0.022	6.250 ± 0.020	6.343 ± 0.015	6.223 ± 0.025	6.066 ± 0.034
	R^2^	0.226 ± 0.004	0.289 ± 0.004	0.267 ± 0.003	0.295 ± 0.004	0.330 ± 0.006

The experiment was repeated ten times, and the mean and standard deviation were calculated

### Feature importance

**Fig. 6:**
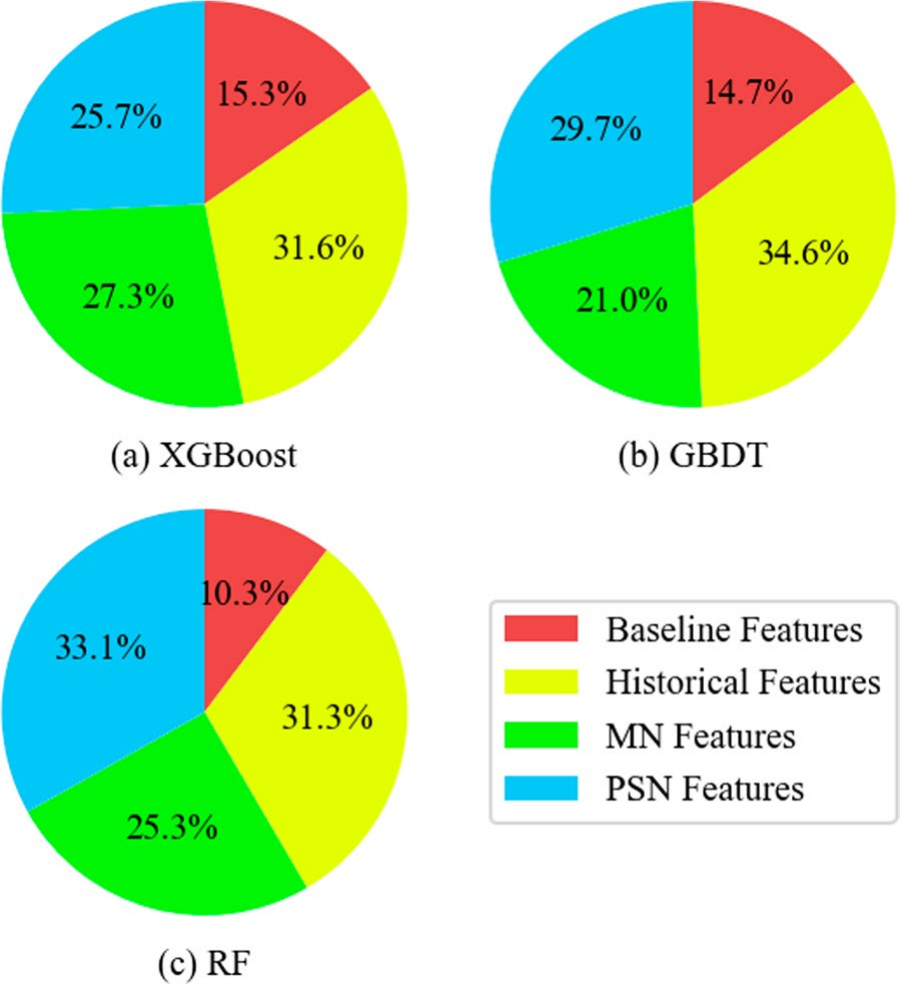
The distribution of feature importance in XGBoost, GBDT, and RF on four feature subset

The feature importance was calculated and all the features aggregated into four disjoint feature subsets by simply summing them up for simplified purposes, as shown in Fig. 6. The historical features have the highest feature importance, closely followed by PSN features. The two kinds of features are both label-related features, where the former utilized the patient’s historical LOS information while the latter used the patient’s neighbors’ LOS information. In addition, the MN features also have relatively high feature importance, representing the disease information at the PoA. To summarize, the ranking of the four feature subsets is: Historical features > PSN features > MN features > Baseline features.

The top ten features for each model are listed in Table 4. Almost all top ten features belong to historical features, MN features, and PSN features. The **mean of the neighbors’ LOS** has the highest feature importance among XGBoost, GBDT, and RF. Some other PSN features, including the **std of neighbors’ LOS**, and the **median of neighbors’ LOS**, are also essential factors. Additionally, the descriptive statistics of historical LOS, such as **mean, median, and maximum**, are important and intuitive factors in predicting the future LOS of patient.

**Table 4:** Top ten features in tree-based models

XGBoost	RI^a^	GBDT	RI	RF	RI
mean of neighbors’ LOS	1	mean of neighbors’ LOS	1	mean of neighbors’ LOS	1
median of historical LOS	0.56	mean of historical LOS	0.74	mean of historical LOS	0.57
max of historical LOS	0.38	last LOS	0.45	median of neighbors’ LOS	0.41
mean of historical LOS	0.28	median of neighbors’ LOS	0.3	median of historical LOS	0.39
LDA-1	0.24	std of neighbors’ LOS	0.25	max of historical LOS	0.16
std of neighbors’ LOS	0.23	last discharge interval	0.21	last LOS	0.14
last LOS	0.21	median of historical LOS	0.19	last discharge interval	0.13
median of neighbors’ LOS	0.2	max of historical LOS	0.19	LDA-1	0.13
last discharge interval	0.17	hospital address	0.16	std of neighbors’ LOS	0.12
LDA-2	0.16	LDA-1	0.13	hospital address	0.1

^a^RI is the relative importance of using min–max normalization. The LDA-1 represents the first component after LDA reduction for network features

### Error analysis

**Fig. 7:**
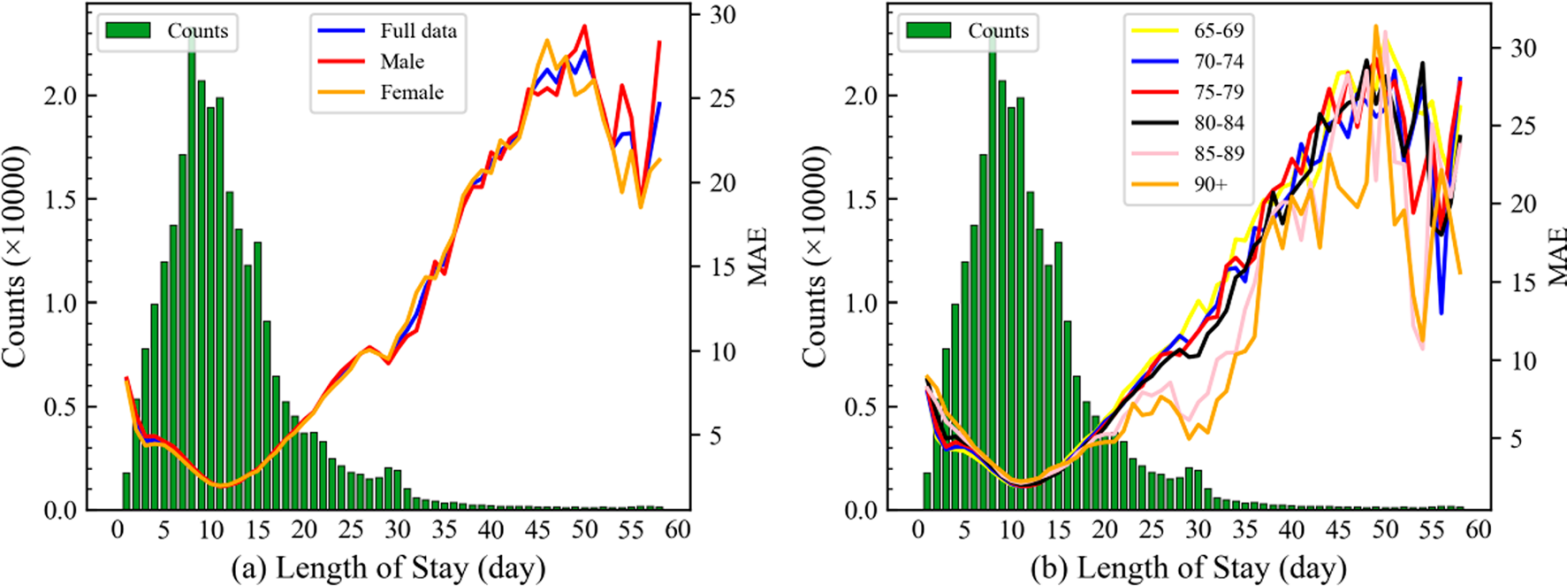
The error distribution of MAE of XGBoost using *Baseline* + *History* + *MN* + *PSN* on the testing set. The green bars are the LOS distributions on the testing set. The results of different subgroups are shown: **a** full data and gender subgroups, **b** age subgroups

The MAE of XGBoost using *Baseline* + *History* + *MN* + *PSN* was 4.024 in the testing set. To explore this model’s predictive ability on different LOS, the MAE of each LOS for several subgroups, such as gender and age, was calculated as shown in Fig. 7. From the full data curve in Fig. 7a, it can be seen that the more hospitalization records for a specific LOS, the smaller is the MAE of the model. The MAE firstly decreases from 8.224 (LOS = 1) to 1.915 (LOS = 11), and then maintains an approximate upward trend until MAE = 24.660 (LOS = 58). When the LOS of a patient is equal to one day, the model’s predictions show a significant deviation. When we investigated the discharge information, which is not available at the PoA, we found that only 53% of patients were discharged from the hospital when medically ordered to leave. The rest might have transferred to another hospital or left the hospital due to lack of money, suggesting that those LOS are hard to predict. For those LOS between 5 and 30 days, this model can make relatively accurate predictions. For the LOS > 30 days, the model has poor predictive ability because the hospitalization records of the LOS gradually decrease and the forecasts of their leaf nodes will be averaged in the decision tree. For example, a sample with LOS = 55 falls into a leaf node, which has five samples with an LOS of 20, 25, 30, 35, and 55, respectively. The prediction value of the leave node is the average of those LOS, which is 33 and accounts for 22 deviations. Due to the hard prediction for small and large LOS, some studies have avoided the problem by regarding the regression task as a classification task such as prolonged LOS (≥ 14) or short LOS (< 14) [44, 45], which has poorer practicability than the regression task. Consequently, these problems still await a direct solution and deserve further exploration.

As regards gender, there is no significant difference between the male and female curves. In contrast, with increases in age, the model’s MAE errors become increasingly smaller when the LOS is longer than 20 days. The older the patient, the more comorbidities they have, resulting in a higher similarity in the PSN, as shown in Fig. 5, which is conducive to improving the model’s performance.

## Discussion

This study has proposed a novel approach to extracting creative and representative network features for early LOS prediction due to the limited data available at the PoA. To the best of our knowledge, this is the first time that features have been extracted from MN and PSN on such a large dataset for LOS prediction.

### Comparison with the other studies of the present literature

Several similar studies that have applied machine learning methods to predict LOS at the PoA are listed in Table 5 for comparison purposes. Due to differences in the data sources, we compare our work with existing studies mainly from the aspects of feature composition and model performance. As shown in Table 5, most existing studies were confined to a single disease and the sizes of their datasets were much smaller than ours, which affects the utility, generalization, and reliability of the model. Moreover, few studies have considered historical features. They tend to calculate only the mean and counts of the LOS of historical records which means that the potential has not been fully realized. We found that other descriptive statistics of historical LOS also impact significantly the LOS predictions as listed in Table 4, such as the median and the maximum. None of the existing studies used the MN and PSN features, which are essential factors in predicting LOS. The historical features, MN features, and PSN features are relatively independent and can enhance model performance from different perspectives, as shown in Table 5. The means of LOS differ from each other, and the truncation strategies for the LOS also vary. Some studies considered the qualified LOS of less than 30 days [17] or truncated at the 98% percentile [46], whereas other studies did not take any action, which accounts for prediction results that are not comparable and vary widely. However, a review study concluded that a model has a strong predictive ability for the LOS if R^2^ > 0.36 [47], which implies that our proposed approach has a superior predictive ability with R^2^ = 0.375.

**Table 5:** Comparison of the results with prior related researches

Study	Condition	Size of dataset	Algorithm	Mean of LOS	Features			Metrics	
					History	MN	PSN	MAE	R^2^
This study	All chronic diseases	1,308,041	XGBoost	12.31	Y	Y	Y	4.024	0.375
Xie et al*.* [8]	All diseases	242,075	RF	None	N	N	N	None	0.15
Liu et al*.* [46]	All diseases	155,474	LR	4.50	N	N	N	None	0.146
Turgeman et al*.* [17]	HF	20,321	Cubist model	None	Y	N	N	1	0.79
Zolbanin et al*.* [48]	COPD	86,338	ANN	5.15	Y	N	N	1.239	0.613
Chang et al. [49]	Ischemic stroke	330	LR	11	N	N	N	None	0.369
Tsai et al*.* [50]	Heart diseases	2377	ANN	5.73	N	N	N	3.76	None

### Limitations and potential future works

The present study has some limitations. First, we adopted zero to fill missing values, which might influence the predictive ability even though the tree-based models are not sensitive about a fill strategy. An appropriate missing value filling strategy, such as *k*-nearest neighbor [51], might achieve better LOS predictions. Second, although we made full use of the historical LOS information, other historical data was not taken into account, such as historical medication use and historical comorbidities. In addition, we used a fixed time window of three years, whereas multi-scale time windows such as going back six months, one year, and three years are likely to be helpful in improving model performance [17]. Third, we extracted the EVC features and disease risk features from the MN to improve prediction accuracy. The potential of the MN can be further excavated, such as network clustering information [28]. Moreover, since the validity of the PSN has been proven by the PSN features, future work could develop a Graph Neural Network (GNN) to use the structural information of PSN, such as Graph SAmple and aggreGatE (GraphSAGE) [52] and Graph Attention Network (GAT) [53], which can construct an end-to-end model by using both network topology information and a node’s feature vectors. In addition, the skewness of LOS results in poor prediction ability of the model when LOS higher than 30 days, as shown in Fig. 7. Some resampling techniques can enhance the number of the tail LOS data, which may bring extra improvement for our proposed methods [54]. Despite these limitations, our proposed approach has sufficient robustness to predict with a certain level of accuracy the hospital LOS for the elderly with chronic diseases at the PoA. Future work will explore the directions indicated above to further improve accuracy.

## Conclusions

This study proposed a novel approach integrating network science with machine learning for making early predictions of hospital LOS in elderly patients with chronic diseases. A space-friendly and high-efficiency development algorithm of MN was presented, making it possible to build MN with millions or even tens of millions of data volumes. The EVC features were extracted from the MN, and the LDA was then performed to reduce the number of EVC features, which can speed up training efficiency and enhance the model performance. Besides, we adopted NMSLIB to construct the PSN to utilize the patient's neighbor's information. The experiment results showed that the network features, could significantly improve model performance across various models. Especially, the R^2^ of XGBoost on *Baseline* + *History* + *MN* + *PSN* was improved by 18.7% compared with the R^2^ of XGBoost on *Baseline* + *History*. To sum up, our proposed approach has enough power to make early LOS prediction for elderly patients, which can offer effective decision support for hospital managers.

## Data Availability

The data that support the findings of this study are available from Health Information Center of Sichuan Province but restrictions apply to the availability of these data, which were used under license for the current study, and so are not publicly available.

## Abbreviations

LOS: Length of stay
PoA: Point of admission
MN: Multimorbidity network
PSN: Patient similarity network
RF: Random forest
ANNs: Artificial neural networks
SVM: Support vector machine
BN: Bayesian network
CCI: Charlson comorbidity index
ECI: Elixhauser comorbidity index
DCN: Disease co-occurrence network
COPD: Chronic obstructive pulmonary disease
Dx2Vec: Diagnoses to vector model
EHR: Electric health record
T2DM: Type 2 diabetes mellitus
PsDF: Patient similarity based on domain fusion
ESKD: End stage kidney disease
AS: Aortic stenosis
LDA: Linear discriminant analysis
XGBoost: Extreme gradient boosting
GBDT: Gradient boosting decision tree
DNN: Deep neural network
MAE: Mean absolute error
RMSE: Root mean square error
R^2^: Coefficient of determination
HDR: Hospital discharge records
RR: Relative risk
UniMP: Unified message passaging model
EVC: Eigenvector centrality
